# Supplementary material for: Content-rich biological network constructed by mining PubMed abstracts
Source: BMC Bioinformatics. 2004 Oct 8;5:147. doi: 10.1186/1471-2105-5-147 (PMC528731; doi:10.1186/1471-2105-5-147)
Supplement: Additional File 5 — The original Chilibot query results of the term "long-term potentiation (LTP)" and 22 other terms, limiting the latest references analyzed to the years 1990, 1995, 2000, and 2004. [file 1471-2105-5-147-S5.bz2 › chilibotAdditionalFile5/ltp2004/html/CREB_PKC.html]

 


 **CREB** and **PKC** 
  
Found 86 abstracts in PubMed,  **30 abstracts were retrieved and analyzed**.  


---

 Search Google  |
 PDF files only 
|  EDU domain only 

---

**Interactive relationship** (e.g. stimulation, inhibition, etc)

- However, MEK1 2 inhibitor, CaMKII inhibitor, and  **PKC**  inhibitor significantly suppressed lead induced  **CREB**  phosphorylation.  Ref: 12832008 Brain Res Bull, 2003
- Mor, F9202, and F9204, which could induce psychological dependence affected via the micro opioid receptor, stimulated intracellular signal pathways involving calcium calmodulin dependent protein kinases CCDPK and protein kinase C  **PKC**  pathways, which in turn initiated  **CREB**  phosphorylation.  Ref: 14653953 Acta Pharmacol Sin, 2003
- These results indicate that lead activated  **PKC** , CaMKII and MEK ERK p90RSK pathways simultaneously, all of which contributed to  **CREB**  phosphorylation.  Ref: 12832008 Brain Res Bull, 2003
- We conclude that lead activates  **PKC** , CaMKII or MEK ERK p90RSK pathways in GT1 7 cells, leading to  **CREB**  phosphorylation and modulation of gene expression.  Ref: 12832008 Brain Res Bull, 2003
- Activation of  **PKC**  with intracaudate injection of 12 O tetradecanoylphorbol 13 acetate mimicked DHPG actions in facilitating the phosphorylation of  **CREB** , Elk 1, and ERK1 2.  Ref: 12220559 Neuroscience, 2002
- **PKC**  inhibition also impaired context and tone dependent fear conditioning and prevented the phosphorylation of Erk 1 2, Elk 1, and  **CREB** , without affecting p90Rsk 1.  Ref: 14739006 Behav Brain Res, 2004
- These results indicate that in neocortical cells in culture,  **CREB**  phosphorylation occurs via Ca, PKA, and  **PKC**  dependent pathways.  Ref: 12421622 Neuroscience, 2002
- Erk 1 2, Elk 1, and  **CREB**  were identified as common targets of PKA,  **PKC** , and CaMK II during memory consolidation the time window and sequence of their phosphorylation was specific for the individual kinase.  Ref: 14739006 Behav Brain Res, 2004
- Furthermore, We found that the MF induced increase of  **CREB**  DNA binding in HL60 cells was dependent on both extracellular and intracellular Ca but not PKA,  **PKC** , ERK, or p38 MAPK by using various pathway inhibitors.  Ref: 12200150 Biochem Biophys Res Commun, 2002
- Inhibition of  **PKC**  with GF109203X significantly attenuated DHPG stimulated  **CREB** , Elk 1, and ERK1 2 phosphorylation.  Ref: 12220559 Neuroscience, 2002

**Parallel relationship** (e.g. studied together, co-existance, homology, etc.)

- Induction of TGF beta 1 synthesis by 30 mmol D glucose was associated with induction of  **PKC**  alpha, phosphorylation of cAMP responsive element binding protein  **CREB**  and activating transcription factor 1 ATF 1, and increased fibronectin and type I collagen translation.  Ref: 12846747 Kidney Int, 2003
- Next, we utilized activators and inhibitors of protein kinase A PKA, protein kinase C  **PKC** , mitogen activated protein kinase kinase MAPKK and calcium calmodulin dependent protein kinase II CaMKII to determine which kinases might be involved in phosphorylating the  **CREB**  in PZHPV 7 cells.  Ref: 12899525 Recent Results Cancer Res, 2003
- The data obtained from this study indicate that selective activation of  **PKC**  is needed for the group I agonist induced  **CREB** , Elk 1, and ERK1 2 phosphorylation in striatal neurons.  Ref: 12220559 Neuroscience, 2002
- Via PKA,  **PKC**  and p38 MAPK dependent signals, dBcAMP also induced inhibitory subunit of NF kappaB IkappaB degradation, IkappaB kinase IKK activation, nuclear translocation of NF kappaB subunit p65 and its association with the  **CREB**  binding protein CBP.  Ref: 14751542 Cell Signal, 2004
- Genes affected in one or the other circumstance include alterations in a 14 3 3 regulator protein of  **PKC** ,  **CREB** , BDNF and GABA receptor subunit proteins, as well as several genes associated with known cannabinoid receptor coupled signaling pathways.  Ref: 12505705 Chem Phys Lipids, 2002
- Taken together, these results indicate that, under primary culture conditions where physiological levels of signaling proteins are present, hCG signals to activate MEK, p42 44 MAPK,  **CREB** , and histone H3 in a predominantly PKA dependent and  **PKC**  independent manner.  Ref: 12130564 Endocrinology, 2002
- We investigated the involvement of hippocampal protein kinase A PKA, protein kinase C  **PKC** , calcium calmodulin dependent protein kinases CaMK II, and mitogen activated extracellular signal regulated kinases 1 and 2 Mek 1 2 in the phosphorylation of their downstream targets extracellular signal regulated kinases 1 and 2 Erk 1 2, cAMP response element binding protein  **CREB** , Elk 1, and p90 ribosomal S6 kinase 1 p90Rsk 1.  Ref: 14739006 Behav Brain Res, 2004
- We also investigated V1aR activation of calcium activated signaling kinases, protein kinase C  **PKC** , calcium calmodulin dependent protein kinase II CaMKII, and the mitogen activated protein MAP kinases MAPK and extracellular signal regulated kinases 1 and 2 ERK1 2, their localization within cytoplasmic and nuclear compartments, and activation of their downstream nuclear target, the transcription factor cAMP response element binding protein  **CREB** .  Ref: 12764111 J Neurosci, 2003
- Our results indicated that cross talk with  **PKC**  and or calcium signaling pathways might sensitize cAMP mediated effects, on  **CREB**  phosphorylation and STC gene expression.  Ref: 12959990 Endocrinology, 2003
- Our findings demonstrate that emodin ameliorates the undesirable effects of concentrated glucose on HPMC via suppression of  **PKC**  activation and  **CREB**  phosphorylation, and suggest that emodin may have a therapeutic potential in the prevention or treatment of glucose induced structural and functional abnormalities in the peritoneal membrane.  Ref: 12846747 Kidney Int, 2003
